# Neonatal Rat Glia Cultured in Physiological Normoxia for Modeling Neuropathological Conditions In Vitro

**DOI:** 10.3390/ijms23116000

**Published:** 2022-05-26

**Authors:** Justyna Gargas, Justyna Janowska, Karolina Ziabska, Malgorzata Ziemka-Nalecz, Joanna Sypecka

**Affiliations:** NeuroRepair Department, Mossakowski Medical Research Institute, Polish Academy of Sciences, 5 A. Pawinskiego Str., 02-106 Warsaw, Poland; jgargas@imdik.pan.pl (J.G.); jjanowska@imdik.pan.pl (J.J.); kziabska@imdik.pan.pl (K.Z.); mnalecz@imdik.pan.pl (M.Z.-N.)

**Keywords:** glial cells, microglia, oligodendrocytes, astrocytes, cocultures, biomaterials, oxygen-glucose deprivation

## Abstract

Cell culture conditions were proven to highly affect crucial biological processes like proliferation, differentiation, intercellular crosstalk, and senescence. Oxygen tension is one of the major factors influencing cell metabolism and thus, modulating cellular response to pathophysiological conditions. In this context, the presented study aimed at the development of a protocol for efficient culture of rat neonatal glial cells (microglia, astrocytes, and oligodendrocytes) in oxygen concentrations relevant to the nervous tissue. The protocol allows for obtaining three major cell populations, which play crucial roles in sustaining tissue homeostasis and are known to be activated in response to a wide spectrum of external stimuli. The cells are cultured in media without supplement addition to avoid potential modulation of cell processes. The application of active biomolecules for coating culturing surfaces might be useful for mirroring physiological cell interactions with extracellular matrix components. The cell fractions can be assembled as cocultures to further evaluate investigated mechanisms, intercellular crosstalk, or cell response to tested pharmacological compounds. Applying additional procedures, like transient oxygen and glucose deprivation, allows to mimic in vitro the selected pathophysiological conditions. The presented culture system for neonatal rat glial cells is a highly useful tool for in vitro modeling selected neuropathological conditions.

## 1. Introduction

Glial cells, playing multiple roles in the central nervous system (CNS), ensure its proper development and functioning under physiological conditions, as well as in many ways react to pathophysiological clues present in the local tissue microenvironment. The glia were shown to be involved in the pathomechanisms of many neurological and neurodegenerative disorders, in which neurons are primarily affected [1]. These include, among others, amyotrophic lateral sclerosis (ALS, in which differentiation of oligodendroglial progenitors are thought to be altered), Alzheimer’s Disease (AD, where microglia seem to play an important role in disease progression), and multiple sclerosis (MS, in which demyelination leads to dystrophy of neurons) [2,3,4,5,6,7,8].

**Figure 1:**
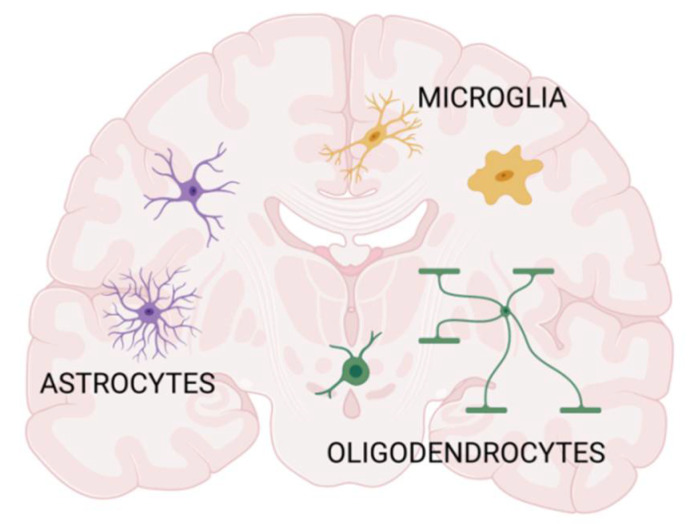
The main types of glial cells of the brain comprise macroglia (astrocytes, oligodendrocytes, and oligodendrocyte progenitors) and microglia. The cells, extracellular matrix, and secreted factors of paracrine signaling participate in intercellular communication and nervous tissue shaping. Figure created with BioRender.com. (accessed on 1 April 2022).

Regarding their ontogenic origin and abundance, glial cells are classified either as macroglia or microglia. Accordingly, macroglia comprise astrocytes, oligodendrocyte progenitors (so-called NG2 cells), and myelinating oligodendrocytes. They originate from neuroectoderm and constitute the most abundant population of cells forming the nervous tissue of the brain and spinal cord [9,10,11]. The microglial cells are derived from the yolk sac and populate the CNS during the restricted period of embryonic development [12,13,14]. The intensive process of gliogenesis in the perinatal and early postnatal period, regulated spatiotemporally by the plethora of the local instructive signals, contributes to developing the unique neural architecture. Each type of specialized glial cells plays a critical role in maintaining CNS development and its proper physiological functioning during the lifespan (Figure 1).

Likewise, astrocytes are mainly recognized as the major suppliers of energy substrates for supporting neurons and signal transduction. They also provide structural support, release axonal guidance molecules and gliotransmitters, promote synaptogenesis and angiogenesis, as well as being engaged in maintaining the blood–brain barrier [15]. Oligodendrocytes are the only cells capable of myelinating nervous fibers within the CNS, ensuring rapid saltatory conduction of the nervous signals [16]. Microglia, although less numerous, are engaged in enhancing proliferation and migration of neural cells, as well as promoting axonal growth during nervous system shaping. Due to the ability to eliminate apoptotic and malformed cells, as well as the excess of progenitors and synapses, microglia contribute to the maturation of neuronal networks [17,18]. In their activated ameboid state, they are also potent to phagocyte cell debris and protein aggregates [19,20,21], contributing to sustaining the local tissue homeostasis. Thanks to constant surveying local tissue microenvironment, microglia play a crucial role in defending CNS from pathogens.

There is, however, an ever-growing list of evidence that all of the enumerated types of glia participate in the immunological response by secreting immunomodulatory molecules [22,23,24,25,26]. Accordingly, due to sensing external stimuli, all of the glial cell types are able to release active compounds, acting as instructive signals for neighboring cells and enabling glia–glia and glia–neurons multidirectional intercellular communications. This cellular interplay could be based either on direct cell contacts or could be exerted in a paracrine manner [27,28,29].

Taken together, glial cells are engaged in numerous neurodevelopmental processes (by promoting neurogenesis, expressing guiding clues, and finally shaping the nervous system), support physiological CNS functioning (by providing energy substrates and trophic factors), and modulate tissue response to pathological clues. The regulation of the above-mentioned roles of glia strongly depends on the intercellular cross-talk. However, to learn and describe in detail the mechanisms engaged in various aspects of glia functioning, a reliable and reproducible in vitro model of glia culture is strongly needed. It should be as simple as possible, which means not including factors that could potentially influence the investigated processes.

The protocols routinely used for cell culturing usually include a spectrum of culture media supplements (like mitogens, morphogens, trophic factors, serum, hormones, etc.) to meet cell requirements. Figure S1 however strongly affects basic cell processes such as survival, differentiation, or secretory profile. Even cell culturing in standard, atmospheric oxygen level (21%), which is a few-fold higher than that physiologically present in the nervous tissue, was shown to significantly decrease cell proliferation and pursue cell differentiation and senescence [30,31,32].

Addressing this issue, we have established in vitro models of rat neonatal glial cells cultured in physiologically normoxic conditions (i.e., 5% O_2_), in the culture media of the minimal, restricted composition, either as monocultures (of astrocytes, oligodendrocytes or microglia) or cocultures of the chosen types of glial cells (microglia-oligodendrocytes, microglia-astrocytes, etc.). The models have been tested and gradually improved over the past decade. The presented in vitro restricted glial models could be useful for investigating basic mechanisms of cell response and intercellular cross-talk, modeling selected neonatal disorders (like hypoxia, hypoxia-ischemia, hypoglycemia, infections, inflammation), testing neurodevelopmental neurotoxicity or new therapeutic screening [33,34,35,36,37].

## 2. Results

**Figure 2:**
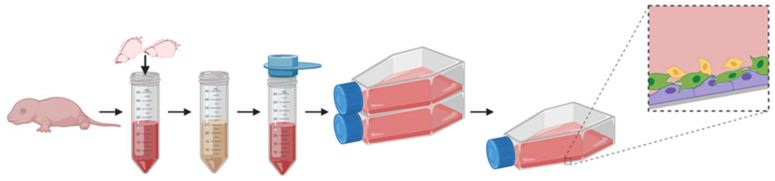
The scheme of establishing primary mixed glial cells from the brains of neonatal rats for the purpose of obtaining the three main types of glial cells. Figure created with BioRender.com. (accessed on 1 April 2022).

**Figure 3:**
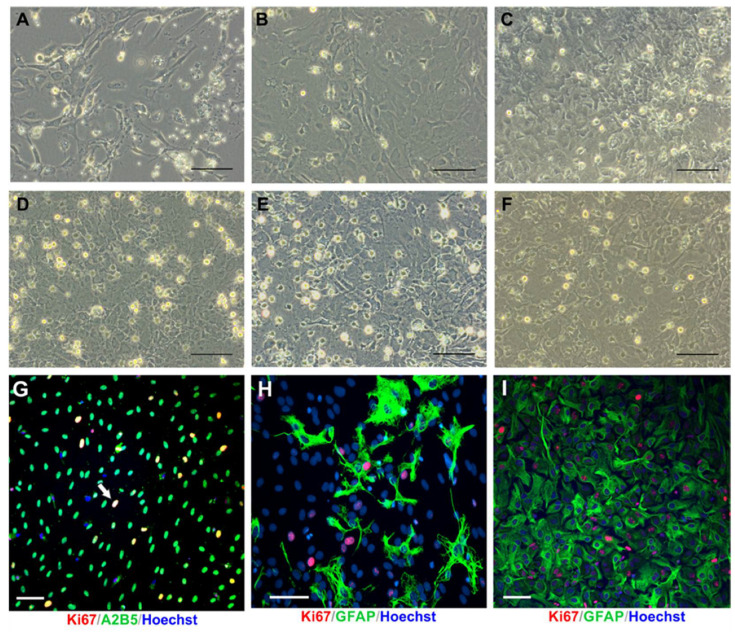
The primary mixed glial culture established from neonatal rat brain hemispheres. (**A**) Attached astrocytes (long, fibroblastic-like morphology) together with microglia and OPCs (round, opalescent cells) on 4 DIV; (**B**) Semi-confluent mixed glial culture on 6 DIV; (**C**) Increase in a number of round cells on a top of astrocyte layer on 8 DIV; (**D**) Constant increase in microglia/progenitor cells in confluent primary culture on 10 DIV; (**E**) Mixed glial culture before separating cells by shake-off method on 11 DIV; (**F**) The primary culture after detaching microglia (for 1 h) and OPCs (for 15–20 h); the separating of oligodendroglial progenitors could be repeated with aim of increasing efficiency of cell isolation; (**G**) Immunolabelled dividing cells (Ki67^+^, red) and A2B5^+^ (neural progenitors, green) 24 h after cell seeding; (**H**) Primary glial cultures, stained with anti-GFAP antibody (green) to visualize differentiating astrocytes and anti-Ki67 antibody (red) to indicate proliferating cells on 4 DIV; (**I**) Confluent astrocyte layer (GFAP^+^, green) and dividing glial cells (Ki67^+^, red) on 8 DIV. Cell nuclei are stained with Hoechst (blue). Scale bar corresponds to 100 μm.

The primary cell cultures were derived from the brain hemispheres of neonatal rats. Between 24 and 48 h postpartum, the average body mass of rat pups was approximately 7 g, while the weight of the brain corresponded to 261.53 mg, thus constituting about 3.7% of the total body mass in this very early postnatal period (Figure 2). Establishing the mixed glial cultures allows obtaining three types of glial cells at the same time: two types classified as macroglia (astrocytes and oligodendrocyte progenitors of ectodermal origin), and microglia, which originates from mesoderm and populates the forming brain during early embryogenesis. Seeded as a single cell suspension, the cells proliferated and differentiated (Figure 3A,B), developing a layer of astrocytes with OPCs and microglia scattered on their surface (Figure 3C–F). The mixed glial culture expressed characteristic neural markers, including A2B5 and GFAP, as well as Ki67 for dividing cells (Figure 3G–I). Thanks to differential adhesive properties, the particular cell fractions could be separated by the prolonged shaking-off method, originally described by McCarthy and de Vellis (1980) [38] as a method for establishing astroglial and oligodendroglial cell cultures. Indeed, our studies on glial cells indicate that neonatal oligodendrocyte progenitors exhibit poor adhesive features in comparison to the other fractions used in experiments and therefore slowly adhere to uncoated glass or plastic surfaces. This observation could be useful for further purification of glial populations, up to nearly 99% of homogeneity. Moreover, the additional step during the cell detachment procedure based on repeating the overnight OPC shaking-off allowed to increase the efficiency of cell separation by 55%, as calculated from the entire number of obtained oligodendroglial progenitors.

### 2.1. The Glial Cells of Rat Neonatal Brains

The number of glial cells of particular cell fractions derived from rat neonatal brains after 12 DIV in mixed glial cultures (without the addition of mitogens) was estimated as 85.84 × 10^5^/brain for astrocytes, 26.52 × 10^5^/brain for OPCs, and 5.77 × 10^5^/brain for microglia. Accordingly, the subsequent calculations indicated that the astrocytes constitute up to 72 ± 10% of the cultured neonatal glial population, while the percentage of OPCs and microglia was estimated at 20.87 ± 8.7% and 7.19 ± 3.17%, respectively.

To mimic physiological conditions typical for the CNS, the obtained cells were kept in an atmosphere of 5% oxygen, relevant for the physioxia of the nervous tissue. To study selected cell processes and cellular interactions, the glia were cultured up to 5 DIV in minimal, serum-free media to avoid stimulation by added active factors, which might potentially affect the studied mechanisms.

**Figure 4:**
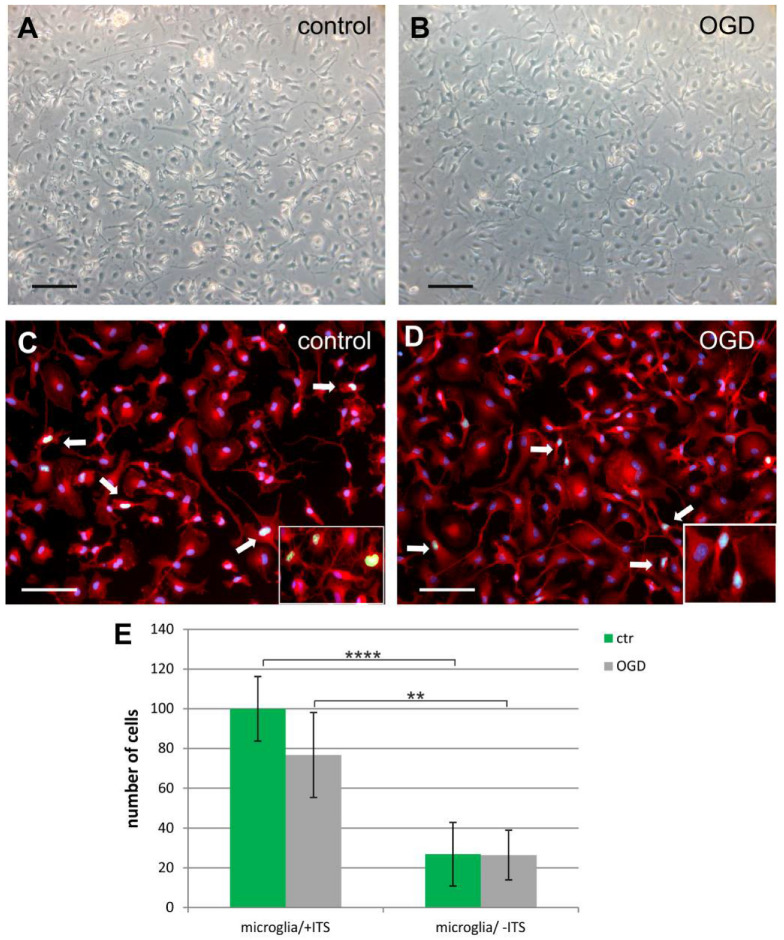
Microglia, the first cell type obtained from the mixed primary culture. (**A**) Live imaging of microglia cultured in serum-free condition in physiological normoxia, 24 h after seeding; (**B**) Live imaging of microglia at 24 h after the OGD procedure; (**C**) During in vitro culturing, the typical morphology of resting microglia is retained, as confirmed by immunostaining with IBA1 marker (red). During the first 2 DIV, cells actively proliferate, as indicated by labelling IBA1^+^ cells with anti-Ki67 antibody (green). (**D**) Labelling microglia 24 h after OGD procedure shows no significant changes in cell morphology and the rate of proliferation. White arrows indicate proliferating Ki67^+^ microglia. Cell nuclei are visualized with Hoechst (blue). Scale bar corresponds to 100 μm. (**E**) Graphical presentation of the ITS influence on microglial culture density on 2DIV in controls and after OGD. Serum-free culture medium is conducive for neonatal microglia culturing in physiological normoxia, however ITS addition significantly increases culture density. ** *p* < 0.01; **** *p* < 0.0001.

Microglia, the first cell population obtained during the detachment procedure, were characterized by typical in vitro morphology (Figure 4A,B) and expressed characteristic lineage-specific markers of inactivated phenotype, i.e., IBA1 (Figure 4C,D) and OX-42 (known also as anti-CD11b or Anti-Integrin αM). This glial population proliferated in vitro, as deduced from the staining of cell nuclei with an antibody directed against Ki67 (Figure 4C,D). Serum-free culture medium was conducive for sustaining microglia in vitro in physiologically normoxic conditions, however ITS addition significantly increased culture density, as calculated on 2 DIV (Figure 4E).

**Figure 5:**
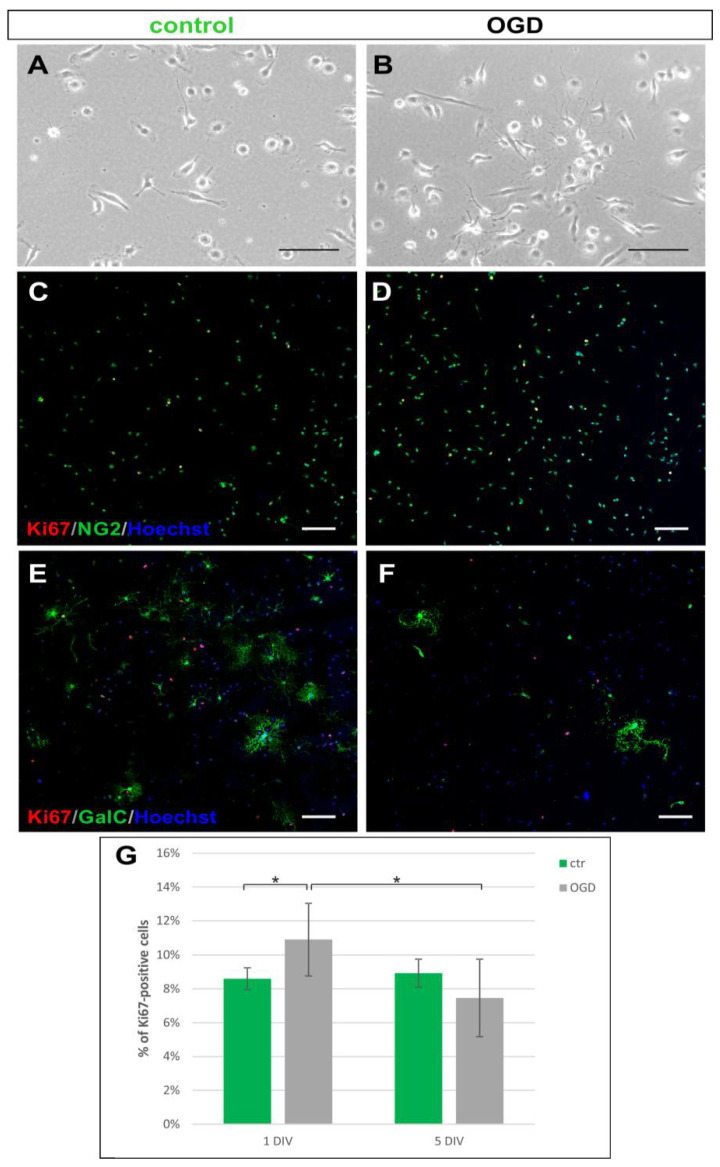
Proliferating and differentiating neonatal oligodendrocytes in vitro. Live imaging of oligodendrocytes cultured in the serum-free condition in physiological normoxia (**A**) and 24 h after OGD procedure (**B**). (**C**,**D**) Dividing, as indicated by immunostaining with anti-Ki67 antibody (red), NG2-positive oligodendrocyte progenitors (green), which are more numerous after the OGD procedure. (**E**,**F**) GalC-positive (green) differentiating, ramified cells in proliferating (Ki67^+^) oligodendrocyte population. Temporal limitation of oxygen and glucose exerts a negative effect on cell branching during the maturation process. Cell nuclei are visualized with Hoechst (blue). The scale bar corresponds to 50 μm. (**G**) Graphical presentation of the oligodendrocyte number in controls and OGD-subjected cultures. OGD procedure stimulates cell proliferation during the first 24 h in physiologically normoxic conditions in vitro. * *p* < 0.05.

Oligodendrocyte progenitors, detached as a middle fraction of glia isolation, proliferated and differentiated in vitro in physiologically normoxic conditions, elongating branched cell processes (Figure 5A,B). The immunofluorescent labeling with a panel of standard, cell-lineage specific markers (NG2, CNP, GalC), specific for the subsequent stages of oligodendrocyte differentiation, enabled the visualization of the maturing cells extending cell processes of complex morphology (Figure 5C–F). Subjecting OPCs to the OGD procedure stimulated cell proliferation from 8.6 ± 0.6% Ki67+ controls to 10.9 ± 2.1% of dividing, Ki67-positive OPCs in the OGD-subjected cultures as calculated 24 h after the insult (*p* < 0.001) and from 6.876 ± 3.03% to 8.93 ± 1.8% as estimated 48 h after OGD (*p* < 0.05) (Figure 5G). Oligodendrocytes cultured in serum-free medium in physiologically normoxic conditions continued to divide, however, the proliferation of control cells decreased from 8.6 ± 0.6% on 1st DIV to 6.39 ± 1.1% on 3rd DIV (*p* < 0.01).

Astrocytes, cultured as confluent cell layer and detached as the third cell fraction, were seeded on the 6-well plates. The larger surface area supported cell culturing for a prolonged time, up to 7 DIV. After the OGD procedure, performed to simulate in vitro the hypoxic-ischemic insult associated with perinatal asphyxia and neonatal stroke, the number of astrocytes varied in regard to the biomolecules used for cell attachment.

### 2.2. Effect of Cell Culturing on Biomaterials

To verify if the components resembling physiological cell microenvironment exert any effects on cell culturing in vitro, the cells obtained from neonatal rat brains (n = 33) were seeded on the surfaces coated with one of the selected compounds, including laminin, fibronectin, or ECM gel (mimicking extracellular matrix composition and containing among others laminin, collagen type IV, heparin sulfate proteoglycan, and entactin).

**Figure 6:**
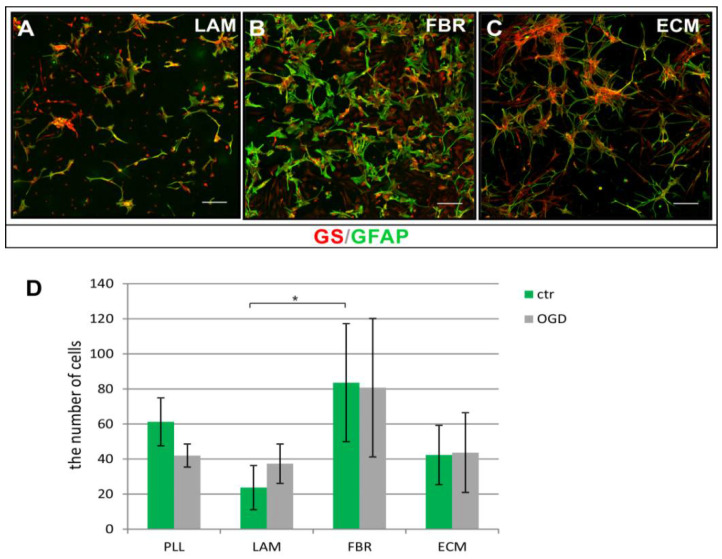
Neonatal rat astrocytes (GS^+^/GFAP^+^) cultured on surfaces covered with different biomolecules: laminin (**A**), fibronectin (**B**), and Extracellular Matrix components (**C**). Culture density depends on diversified adhesive properties of the applied biomaterials and is the highest when astrocytes are cultured on fibronectin. Cell nuclei are stained with Hoechst dye (blue). Scale bar represents 50 μm. (**D**) Number of cultured astrocytes in regard to molecules used for coating culture surfaces. * *p* < 0.05.

Effects of cell adhesion to the applied compounds were evaluated 24 h after cell plating in the context of the cell number and expression of classical astroglial lineage-specific markers. Accordingly, the biomolecules used for coating culture surfaces were shown to highly affect astrocyte numbers (Figure 6A–C). While cell number was similar when astrocytes were cultured on either PLL, LAM, or ECM, the increase by approximately 36.4 % in culture density in comparison to PLL was observed when cells were attached to fibronectin (Figure 6D).

**Figure 7:**
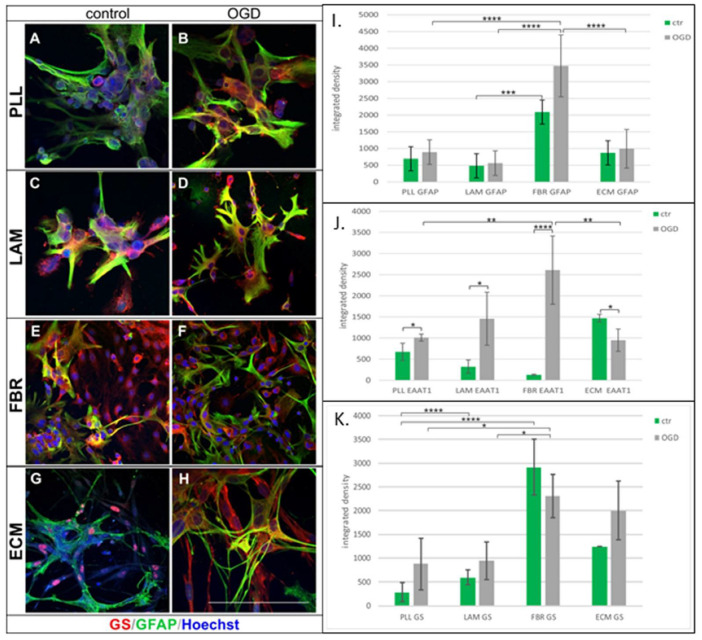
Influence of biomolecules used for astrocyte attachment on the expression of classical astrocyte markers: GS, GFAP, and EAAT1. Double immunocytochemical labelling of astrocytes (GS^+^/GFAP^+^) cultured on surfaces coated either with PLL (**A**,**B**), Laminin (**C**,**D**), Fibronectin (**E**,**F**), or ECM gel (**G**,**H**). Cell nuclei are stained with Hoechst dye (blue). Scale bar represents 50 μm. (**I**) Analysis of GFAP marker expression in regard to biomaterial used for cell culturing; (**J**) Measured EAAT1 expression by astrocytes attached to various biomolecules. (**K**) GS expression depends on the biomolecule used for coating the culture surface. * *p* < 0.05, ** *p* < 0.01; *** *p* < 0.001, **** *p* < 0.0001.

As revealed by the obtained results, the adhesive properties of the compounds used for coating culturing areas turned out to exert a significant impact on the expression of specific markers of astrocyte lineage. GFAP, which is a cytoskeleton component belonging to the type III intermediate filaments, was the first marker analyzed by means of measuring the fluorescence intensity (Figure 7A–H).

Accordingly, while there were no significant differences between control and OGD-subjected cells attached to PLL, the GFAP expression increased significantly (*p* < 0.0001) after OGD when the cells were cultured on fibronectin by approximately 390% (versus PLL) and by 350% in comparison to cells cultured on ECM (Figure 7I). The differences in marker expression between cells cultured on various biomaterials were also highly pronounced in the case of EAAT1 examination. Antibody directed against EAAT1 stained excitatory amino acid transporter 1 (engaged in the transport of glutamic and aspartic acids) and was shown to be upregulated after OGD by 150% for cells cultured on PLL, by 220% on LAM, and as much as by 2000% on FBR (Figure 7J). Another classical marker of astrocytic lineage corresponds to glutamine synthetase (GS) involved in glycogen metabolism. This enzyme immunolabelling increased by approximately 1033% (*p* < 0.0001), as compared to PLL, when cells were attached to fibronectin and by 710% in cells cultured on the surface coated with ECM (Figure 7K). The results are summarized in Table 1.

### 2.3. Cocultures of Glial Cells

The isolated fractions of particular cell types can also be used for coculture experiments for studying cellular interactions, either in direct cell-to-cell contacts or via paracrine signaling.

**Figure 8:**
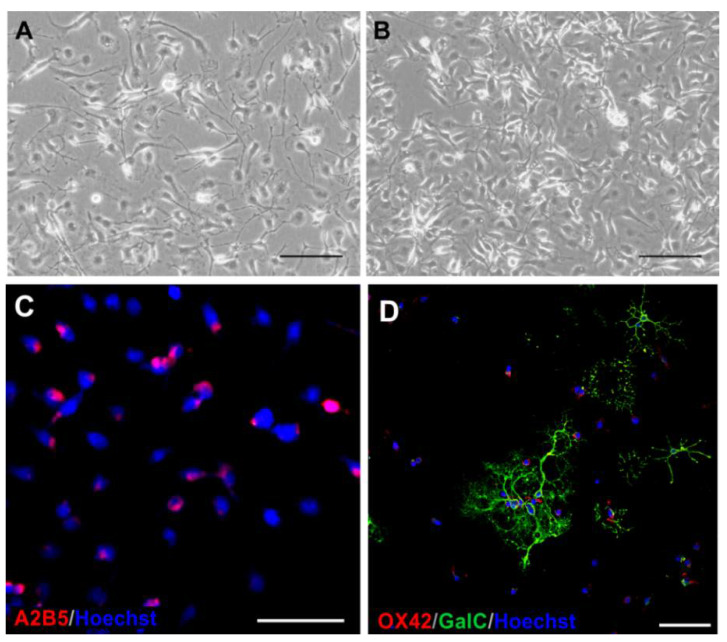
Coculture of rat neonatal oligodendrocytes and microglia. (**A**) Live imaging of control coculture; (**B**) Live imaging of coculture subjected to OGD procedure; (**C**) Immunostaining of cocultured cells with A2B5 antibody (red) allowing to distinguish neural cells, represented by oligodendrocytes. Microglia of mesenchymal origin remains unstained. (**D**) Differentiating, GalC-positive oligodendrocytes (green) cocultured with microglia, immunolabelled with OX42 marker (red). Cell nuclei are stained with Hoechst dye (blue). The scale bar is an equivalent to 50 μm.

**Figure 9:**
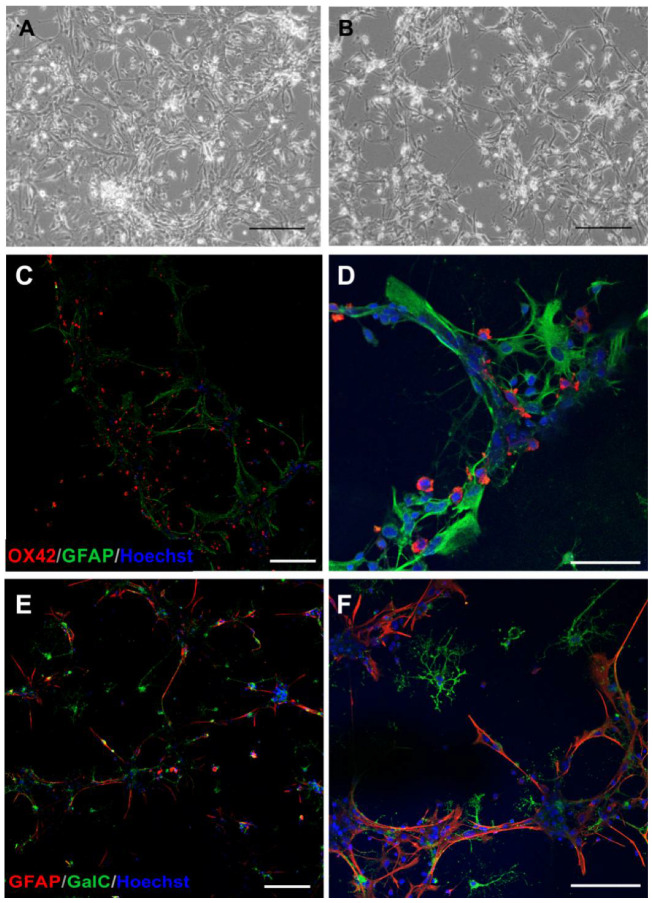
Coculture of rat neonatal astrocytes with either microglia or oligodendrocytes. (**A**) Live imaging of control astrocyte-oligodendrocyte coculture; (**B**) Live imaging of coculture subjected to OGD procedure; (**C**,**D**) Immunostaining of microglia-astrocyte coculture stained with specific antibodies to distinguish the type of cultures cells: OX42 for microglia (red) and GFAP for astrocytes (green); (**E**,**F**) Astrocytes (GFAP^+^, red) cocultured with differentiating GalC-positive (green) oligodendrocytes. Cell nuclei are stained with Hoechst dye (blue). The scale bar is an equivalent of 50 μm.

The former is based on plating cell fractions in various proportions on the same culturing surface, while the latter method requires the application of inserts to separate particular cell types. Culturing cells on the inserts plated into wells allows us to perform studies on cell communication via active factors released to the local milieu. In our experiments, the direct oligodendrocytes-microglia (Figure 8A–D), oligodendrocytes-astrocytes (Figure 9A,B,E,F), and astrocytes-microglia (Figure 9C,D) cocultures were established.

Since the applied basic conditions, such as coating culture surface with PLL and serum-free medium without supplements, except for ITS, allowed for culturing all three examined cell types up to 7 DIV, the same conditions were also applied for assembling glial cocultures. By application of panels of the lineage-specific markers, the cell fractions could be distinguished and further studied in the context of their basic processes, e.g., morphology, survival, and progress in differentiation. Moreover, assembling different experimental variants cocultures of control, control plus OGD-treated, or OGD-subjected cell fractions allows to us extend studies on evaluating intercellular communications and interdependence.

## 3. Discussion

Studies on cell response to extracellular clues present in the local microenvironment in vitro require defined, strictly controlled conditions to avoid the presence of active factors that could potentially modulate elucidated processes. The mixed primary cultures of neonatal glial cells offer an attractive possibility to obtain the three most important glial fractions at the same time, from the same neonatal rat brains. The shaking-off method based on sequential detaching of particular fractions of glial cells is economical and allows to reduce the number of animals required for scientific research. Importantly, the extended in vitro basic and pre-clinical studies are in line with the 3Rs (replacement, reduction, and refinement) principle, allowing for replacing a part of in vivo experiments on living animals with alternatives offered by in-depth research on a spectrum of in-a-dish culture/coculture systems.

Comparative studies on different types of neonatal glial cells are especially useful in evaluating physiological processes during CNS development, cell response to pathological clues present in cell environment, as well as mutual interactions between particular types of glial cells. Besides their well-recognized functions like providing energy and trophic support [39], myelination of axons guarantees fast and efficient nervous signal propagation and keeping tissue homeostasis [40,41,42], and glial cells are thought to be actively engaged in shaping the nervous system. There is an ever-growing list of evidence that the interactions between different types of glial cells play crucial roles in developing CNS.

Likewise, the defined subset (CD11c^+^) of neonatal microglia was postulated to stimulate myelinogenesis [43,44]. Interactions between glial cells were also shown to be engaged in the modulation of mechanisms associated with neuroinflammation and other pathophysiological stimuli [45,46].

The possibility of obtaining the three types of glial cells from the same mixed glial culture enables establishing the needed cocultures for elucidation of the dynamic interactions between glial cells and extending the performed studies by comparative studies on glial monocultures. The main advantage of the described protocol is the possibility of performing studies on the naïve cells in the primary cultures which much more reflect physiological cell response in comparison with the immortalized cell lines. Accordingly, the mouse retroviral immortalized BV-2 and N9 cells were shown to not fully adjust to microglia characteristics [47,48] which limits the credibility of the obtained results. Other clues potentially modulating investigated mechanisms are associated with active compounds added to culture media to support cell culturing. One of the potent supplements is serum, rich in trophic ingredients (yet of undefined, varying composition), which was shown to significantly affect microglial gene expression [49]. Keeping cells in defined serum-free conditions turned out to be effective in the presented study on neonatal microglia, as well as in the case of microglia obtained from brains of adult animals [50]. Cultured neonatal cells were stained with classical microglial markers and acquired typical ramified morphology. Modulating cell properties by supplementing the culture medium with the selected active factors is, however, a very useful tool to promote microglia polarization between proinflammatory M1 and anti-inflammatory M2 phenotype [51,52]. Likewise, polarization towards M1 microglia could be achieved for instance by stimulating cells with IL1β or IFN-γ, while enhancement of conversion into M2 phenotype could be enhanced by treating cells with either IL-4, IL-10, or IL-13 [53,54,55]. These tools allow designing extended studies on microglia and their cocultures with other cell types to enrich our understanding of microglia biology and cellular interplay.

Isolated oligodendrocyte progenitors undergo a multistage process of differentiation, terminated by gaining myelinogenic potential. Maturing oligodendrocytes elaborate multibranched cell processes, which might form myelin segments around axons. Each stage of cell differentiation can be identified by a panel of specific markers, including antibodies directed against unique myelin components [16,56]. OPC development in vitro recapitulates physiological process, and cell culturing in serum-free conditions might be further supported by the addition of a defined supplement composed of insulin, transferrin, and sodium selenite to enhance myelinogenesis, which might be valuable if the studies on the process of myelination are planned in cocultures with neurons. Neonatal OPCs are known to be extremely sensitive to the influence of external signals [57]. As proven by our studies, even short deprivation of glucose and oxygen, imitating in vitro hypoxic-ischemic insult exerts a negative impact on OPC survival and differentiation. Considering these observations, the presented protocol serves as an important tool for studying OPC biology and testing potential treatments to develop an effective pharmacological therapy for white matter disorders resulting from perinatal asphyxia or neonatal stroke.

The minimal, serum-free, and strictly defined medium was also shown to be supportive for neonatal astrocyte culturing, which expressed classical lineage-specific markers, like GFAP, the type III intermediate filaments, known as cytoskeleton components involved in shaping astrocytic structure and morphology [58,59]. Other examined characteristic astrocytic markers included EAAT1 (known also as glutamate aspartate transporter 1, GLAST-1) and GS, which engaged, among others, in the transport of excitatory amino-acid and glycogen synthesis and use [60,61,62,63]. With aim of creating in vitro microenvironment mirroring physiological conditions in many aspects, the biomaterials corresponding to extracellular matrix components were applied for cell culturing. In the case of astrocytes, cell plating on surfaces coated with biomaterials was shown to exert a significant impact on both the cell morphology and expression of characteristic astrocytic markers. The presented findings are in line with the previous observations that fibronectin played a crucial role in astrocyte adhesion and shaping [64]. There is a growing recognition that ECM components are engaged in nervous tissue shaping during development, as well as in the formation, together with glia, of synapses [65,66,67,68]. In this context, the model using ECM components creates new opportunities to extend studies of selected processes during neurodevelopment. Thus, neonatal astrocyte culturing might be useful for studying cell biology in conditions mimicking physiological microenvironment. These observations might be also valuable when planning disease-in-a-dish modeling, especially in the case of coculture experiments [69,70,71].

Establishing cocultures is an additional advantage to evaluate selected mechanisms of crosstalk between cells constituting neural tissue. Intercellular communication is essential for sustaining local homeostasis, neurodevelopmental processes, and tissue response to pathophysiological events [70,72,73,74,75]. The interplay between particular types of cells constituting the nervous tissue is crucial for overcome in inflammatory processes associated with disbalance in local microenvironment composition, promoting the initiation and enhancing neuroreparative processes [76,77]. In vitro establishment of cocultures with the various proportion of particular cell types allows for mimicking the physiological cellular composition of the nervous tissue. Modulating cell proportion or their activation state (e.g., coculturing cells with microglia or astrocytes activated by various stimuli) is especially useful in creating in vitro models of various pathogenic conditions, evaluating processes triggered as cell response to the presence of pathological clues, as well as test potential treatment options [78,79,80,81].

Reassuming, the presented protocol allows for obtaining and culturing three fractions of neonatal glial cells in minimal, strictly defined, serum-free conditions, in physiological normoxia. Glia can be cultured as separate fractions or cocultures to evaluate cell mechanisms or unravel mutual intercellular communication. Elimination of media supplements that might potentially influence the studied biological processes increases the credibility and translational potential of the obtained results. The presented protocol can be used for evaluating basic cell processes and to study how the cells react to pathophysiological conditions. One of the main advantages of the protocol is the possibility to investigate interactions of neonatal glial cells during both physiological processes of nervous tissue formation and their interplay in response to stimuli present in the local microenvironment [45,82,83,84,85].

The protocol is suitable for in-a-dish modeling of various conditions by modifying the milieu of cultured cells. A spectrum of pathophysiological cases can be imitated in vitro, considering clinical needs in neonatal healthcare. Accordingly, as shown in the present study, the OGD procedure reflected the hypoxic-ischemic insult, associated with perinatal asphyxia or neonatal arterial ischemic stroke [86,87,88,89]. To mimic in vitro transient or chronic hypoglycemia, the standard culture medium is replaced by that with a reduced glucose concentration (e.g., DMEM with lowered glucose content: 1000 g/mL instead of 4500 g/mL; ThermoFisher, Waltham, MA, USA). Neuroinflammation (as a result of for instance infection) could be imitated in vitro by treatment with various concentrations of bacterial lipopolysaccharide (LPS), tumor necrosis factor α (TNFα), or Ureaplasma species [90,91,92,93]. Glial cell monocultures or cocultures could be also used to mimic different postnatal conditions, like for instance hypernatremia, hyperchloremia, hypo/hypercalcemia, etc. [94,95]. The neonatal glial cultures are also useful for studies on neurodegenerative diseases, including PD [96,97], AD [98,99,100,101], MS [102,103], or triggered by exposure to environmental toxicants and pollution [104,105,106]. Culturing cells in oxygen concentration is relevant for that present in the nervous tissue, and the elimination of serum and other supplements create opportunities to eliminate microenvironmental clues, potentially affecting investigated processes or modulating drug effectiveness.

The culture system could be used also for drug testing, the development of new treatment options, or as extended pre-clinical trials to elaborate new pharmacological strategies. The main advantage of the culture system is a possibility to verify the tested compound effect on particular cell types in comparison to cocultures, mimicking a neural microenvironment.

## 4. Materials and Methods

### 4.1. The Primary Mixed Glial Culture

Neonatal Wistar rats (24–48 h postpartum, *n* = 108) were mildly introduced into deep hypothermia and decapitated, according to the procedure approved by the II Local Ethics Committee on Animal Care and Use. After removing the meninges, both cerebral hemispheres were isolated and mechanically dispersed 10–15 times with a Pasteur pipette and then by a 1.2 mm Luer-Lock needle, in a solution composed of Dulbecco’s Modified Eagle’s Medium (DMEM, high glucose, GlutaMAX Supplement, Gibco), 1% antibiotic-antimycotic solution (AAS, Sigma-Aldrich, St. Louis, MI, USA), and 10% fetal bovine serum (FBS, heat-inactivated, Gibco). The resulting homogenate was passed through a 40-μm Cell strainer (pluriSelect) to obtain a purified single-cell suspension, and seeded on poly-L-lysine hydrobromide (PLL, Sigma-Aldrich) coated bottles with a 75-cm^2^ culture area (Thermo Scientific Nunc, Waltham, MA, USA) in a density of 5 × 10^4^ cell/cm^2^ and kept in vitro for the following days as a mixed primary culture of glial cells. Culture conditions were fixed for 37 °C, 5% CO_2_, 21% O_2_, and the culture medium was changed every 2–3 days. After 11–12 days in vitro (DIV), when the cultures become confluent, the individual glial fractions (corresponding to microglia, oligodendrocyte progenitor cells (OPCs), and astrocytes, respectively) were isolated in regard to their diversified adherence proprieties (Table 2). Isolated cell fractions were then kept in serum-free conditions up to 7 DIV under normoxic conditions in a cell incubator dedicated to cell culturing in the regulated oxygen concentration with a solid Zirconia O_2_ sensor to precise control of physiological oxygen levels to simulate in vivo conditions (The IncuSafe MCO-170 M Multigas Incubator, PHCBI). The parameter corresponding to oxygen concentration was fixed at 5% and the oxygen excess was eliminated by nitrogen flux.

### 4.2. Isolation of Microglia

Microglia was the first cell population detached from the original primary mixed glial culture. Accordingly, after 11–12 DIV, the flasks were placed on the orbital shaker (Biosan) and inserted into a cell incubator with a stable temperature of 37 °C. Rotation was settled for 160 rpm. After 1 h of shaking, the medium was collected and centrifuged (5 min, 2500 rpm), while the fresh medium was added to the cells remaining in culture flasks. The resulting pellet containing the microglial cells was resuspended in the serum-free medium composed of DMEM, 1% AAS, and supplemented with 1% the Insulin-Transferrin-Selenium-A Solution (ITS) (Invitrogen, Waltham, MA, USA). Cells were counted with the use of Bürker’s chamber, seeded at a density of 4 × 10^4^ cells/cm^2^ on either 6- or 24-well plates (Thermo Scientific Nunc) coated with poly-L-lysine to facilitate cell adherence, and cultured in physiologically normoxic conditions (5% O_2_, 37 °C, 5% CO_2_).

### 4.3. Separation of Oligodendrocyte Progenitors

After detaching microglia from the top of the mixed primary cultures, flasks were mounted again on the orbital shaker for another 15–20 h (160 rpm, 37 °C) to separate oligodendrocyte progenitors from the remaining astrocyte layer, tightly adhered to the flask bottom. As in the case of microglia, the culture medium was collected and spun down (5 min, 2500 rpm). The fresh medium was added to the flask, while the pellet was dispersed in a serum-free medium. The same medium was used for culturing all of the isolated glial cell fractions. Separated OPCs were seeded at a density of 2 × 10^4^ cells/cm^2^ on 6- or 24-well (Thermo Scientific Nunc) poly-L-lysine coated plates. Before any further experiments, OPCs were allowed to attach to culture plates for 4 h due to their low adherence properties. To increase the efficiency of OPC isolation and the homogeneity of the remaining astroglial fraction, the entire procedure was repeated. To increase the monoculture homogeneity, the cell suspension could be additionally seeded into uncoated Petri dishes (or culture flask) for 20–30 min, with gentle shaking. Due to different adhesive properties, the astrocytes or microglial cells will sediment and adhere to the surface of the dish, while OPCs stay in the suspension or are only loosely attached to the uncoated plastic or glass surface. Gentle tapping or washing with culture medium/PBS allows easily detaching OPCs while contaminating astrocytes or microglia remains attached. The supernatant with OPCs is then transferred to another dish as the OPCs population of the increased homogeneity. During the following days of in vitro culturing in physiologically normoxic conditions (5% O_2_, 37 °C, 5% CO_2_), OPCs were differentiated into multibranched mature oligodendrocytes, expressing myelin components.

### 4.4. Detachment of Astrocytes

After collecting microglia and oligodendroglial progenitors, the astrocyte layer was subjected to mild trypsinization (5 min, 37 °C, Trypsin-EDTA (0.05%), phenol red, Gibco). The procedure was stopped by the addition of FBS (in a 2:1 ratio). Detached cells were centrifuged (5 min, 2500 rpm). The pellet was briefly washed additionally with PBS to remove trypsin. Cells were seeded at a density of 4 × 10^4^ cells/cm^2^ on either 6- or 24-well poly-L-lysine coated plates and cultured in serum-free medium in the physiologically normoxic conditions (5% O_2_, 37 °C, 5% CO_2_).

### 4.5. Cell Counting

The number of cells in each separated glial type was counted in the Bürker chamber with the use of 0.4% Trypan Blue Solution (ThermoFisher). After cell seeding and culturing for 24 h in vitro, the abundance and homogeneity of each fraction were checked by manual counting of cells immunolabeled with lineage-specific markers. Culture homogeneity was estimated by comparison of number of labeled cells with a total number of cell nuclei, stained with Hoechst.

### 4.6. Application of the Selected Biomaterials for Cell Culturing

To create in vitro conditions resembling in many aspects, the physiological microenvironment and the influence of selected biomaterials on basic cell processes (like survival, proliferation, morphology) were tested. Therefore, besides physiologically normoxic conditions for the nervous tissue (5% O_2_) and the application of culture media with the restricted composition, the surface of the plasticware was covered with one of the selected biomaterials. The tested biomaterials included: poly-L-lysine (PLL), fibronectin (FBR), laminin (LAM), and Extracellular Matrix Gel (ECM Gel, Matrigel). PLL as a precursor amino acid was used as a standard plate-cover compound which enables adherence of the cells. FBR is an extracellular matrix component, while LAM is a constituent of the basement membrane. ECM Gel contains various extracellular matrix components, especially different types of collagens. Accordingly, all the plates were coated with PLL and allowed to dry for 24 h. Before cell seeding, wells were then covered with one of the following compounds: fibronectin (Sigma-Aldrich), laminin (Sigma-Aldrich), or ECM Gel (Sigma-Aldrich), diluted in regard to manufacturer instructions and either used immediately for cell plating (ECM) or left for 1 h (Lam, FBR) in room temperature (RT), then washed with warm PBS and used for cell culture.

### 4.7. Cocultures of Glial Cells

The isolated glial fractions could be also assembled as cocultures of two or three types of cells since they could be cultured in the same, serum-free, restricted medium. It should be taken into consideration that the particular cell types exhibit different adherence properties, which is why oligodendrocyte progenitors should be allowed to adhere for at least 2 h. Nonetheless, cells could be cocultured either directly (i.e., with possible cell-to-cell contacts, in semi-confluent density) or indirectly (with an application of cell culture inserts; in this case intercellular communication occurs in a paracrine manner).

### 4.8. Neonatal Hypoxia-Ischemia Model In Vitro

The neonatal glial cultures, developed in vitro in physiologically normoxic conditions and in defined culture media, might be used to model the selected neonatal diseases. To model hypoxic-ischemic insult associated with perinatal asphyxia/neonatal stroke, the oxygen-glucose deprivation (OGD) procedure was applied to the cultured glial cells. Accordingly, the standard culture medium is replaced with the degassed OGD buffer, in which the glucose is replaced with 10 mM mannitol to keep the osmotic concentration unchanged. The temporal limitation of glucose and oxygen was executed in a Modular Incubator Chamber (Billups-Rothenberg) in an environment created by a gas mixture of 95% N_2_ and 5% CO_2_. The OGD duration was estimated at 40 min and was terminated by transferring plates to a standard culture medium and physiologically normoxic conditions. The cultured cells were immunolabelled or collected and stored (in −80 °C) for the subsequent biochemical and molecular analyses.

### 4.9. Identification of Glial Cell Phenotype by Immunostaining

To verify the phenotype of the cultured neonatal glial cells, the immunolabelling with cell-type specific antibodies was performed. Accordingly, the cell monocultures or cocultures were fixed at the selected time points (24 h, referred to also as 1 DIV; 2 DIV, 3 DIV, and 5 DIV) with 4% paraformaldehyde (PFA) for 20 min at room temperature (RT) and washed three times with PBS heated to RT. After short permeabilization with 0.1% Triton X-100 in PBS for 5 min, blocking with 10% normal either goat (Gibco) or donkey (Gibco) serum for 1 h RT was performed, and the selected primary antibodies were applied for the overnight incubation (4 °C). To identify microglia, mouse monoclonal OX-42 antibody (CD11b, 1:200, Abcam, Cambridge, UK) and goat polyclonal IBA1 (1:400, Abcam) antibody were applied. Neural progenitors were labeled with mouse monoclonal A2B5 antibody (1:200, Millipore, Burlington, MA, USA). To determine the stage of oligodendrocyte differentiation, the panel of the lineage-specific antibodies was applied, including mouse monoclonal anti-CNPase (1:100, Merck Millipore, Burlington, MA, USA), as well as rabbit polyclonal anti-NG2 (1:100, Chemicon, Rolling Meadows, IL, USA) and anti-GalC (1:100, Millipore). To distinguish astrocytes, mouse monoclonal anti-GS (1:200, Millipore), as well as rabbit polyclonal antibodies against GFAP (1:200, Millipore,) and EAAT1 (GLAST) (1:200, Millipore), were applied. Proliferating glial cells were visualized with an anti-Ki67 antibody (1:100, Leica, Wetzlar, Germany). After overnight incubation with the enumerated primary antibodies, the cells were washed three times using PBS and the secondary antibody conjugated to fluorescent dyes (either Alexa Fluor-488 or Alexa Fluor-546, in 1:1000 dilution, Thermo Fisher Scientific) was added (1h incubation, RT). After extensive washing cultures with PBS, the cell nuclei were stained with Hoechst 33342 (1:150, Sigma) during 15 min-long incubation (RT). Immunolabelled glial cells were immersed in the Fluoromount™ reagent (Sigma) and used for microscopic analyses and picture acquisition with the use of LSM 780/ ELYRA PS.1 superresolution confocal system (Carl Zeiss, Jena, Germany). The fluorescent intensity of selected markers was quantified using ImageJ software (http://imagej.nih.gov/ij, accessed on 1 February 2022). Accordingly, the captured images were converted to grayscale, and the average intensity was measured after background subtraction.

### 4.10. Statistical Analysis

The immunostained cells were manually counted on randomly selected 8–10 visual fields on each of at least ten slides collected from each of the five experiments. Gathered data were subjected to detailed statistical analysis with the use of dedicated GraphPad PRISM 9.0 software. The one-way analysis of variance (ANOVA) followed by Bonferroni’s multiple comparison test allowed us to compare experimental groups and evaluate the statistical significance of the revealed differences between analyzed probes. For statistical comparison of the differences between two groups, an unpaired two-sample unequal variance Student’s *t*-test was used. The data were expressed as mean standard deviation (±SD) and the differences were taken into consideration as significant if calculated values corresponded to * *p* < 0.05, ** *p* < 0.01; *** *p* < 0.001, **** *p* < 0.0001.

## Figures and Tables

**Table 1:** Effects of different biomaterials on rat neonatal astrocytes in serum-free medium in physiologically normoxic conditions.

	Culture Density	GFAP EAAT1 GS		
PLL	-	-	↑	-
LAM	-	-	↑	-
FBR	↑	↑	↑	-
ECM	-	-	↓	↑

Abbreviations: ↑: increase, ↓: decrease, -: no significant changes.

**Table 2:** Establishing the cultures of particular glial types.

Isolation Methods:		
Microglia	1 h shaking, 160 rpm, repeated	
OPCs	15–20 h shaking, 160 rpm, repeated	
Astrocytes	mild trypsinization, 5 min, 37 °C	
**Cell density [(cell/cm^2^) and % of the cultured neonatal glial population**		
Primary mixed glial culture	5 × 10^4^	n/a
Microglia	4 × 10^4^	7.19 ± 3.17%
OPCs	2 × 10^4^	20.87 ± 8.7%
Astrocytes	4 × 10^4^	72 ± 10%

## Data Availability

Not applicable.

## Supplementary Materials

The following supporting information can be downloaded at: https://www.mdpi.com/article/10.3390/ijms23116000/s1.

## Abbreviations

AAS	antibiotic–antimycotic solution
AD	Alzheimer’s Disease
ALS	amyotrophic lateral sclerosis
CNS	central nervous system
DIV	days in vitro
DMEM	Dulbecco’s Modified Eagle’s Medium
EAAT1	glutamate aspartate transporter 1, GLAST-1
ECM Gel	Extracellular Matrix Gel, Matrigel
FBR	fibronectin
FBS	fetal bovine serum
GS	glutamine synthetase
ITS	Insulin-Transferrin-Selenium-A Solution
LAM	laminin
LPS	lipopolysaccharide
MS	multiple sclerosis
NG2 cells	oligodendrocyte progenitors
OGD	oxygen-glucose deprivation
OPCs	oligodendrocyte progenitor cells
PFA	paraformaldehyde
PLL	poly-L-lysine hydrobromide
RT	room temperature
TNFα	tumor necrosis factor α
