# Supplementary material for: Neonatal Rat Glia Cultured in Physiological Normoxia for Modeling Neuropathological Conditions In Vitro
Source: Int J Mol Sci. 2022 May 26;23(11):6000. doi: 10.3390/ijms23116000 (PMC9180927; doi:10.3390/ijms23116000)
Supplement: Supplementary file 1 [file ijms-23-06000-s001.zip › ijms-1727259-supplementary.pdf]

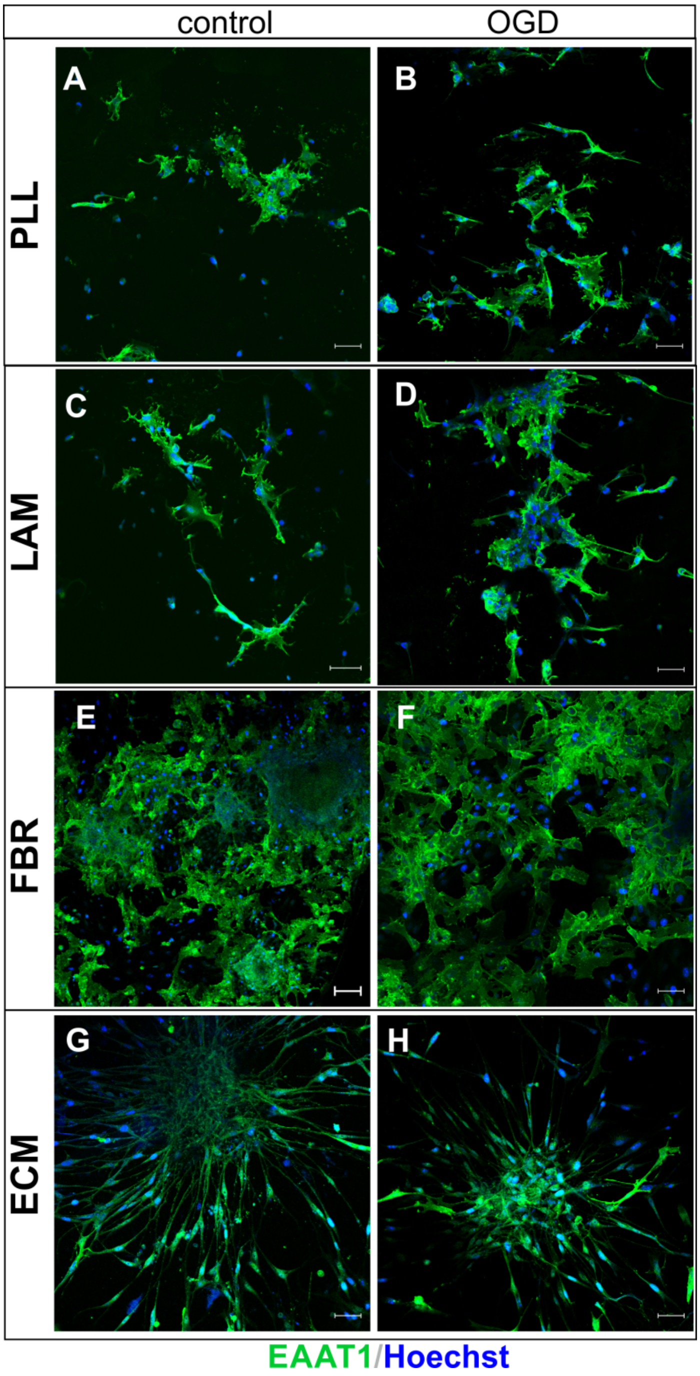

Figure S1. Expression of EAAT1 marker in neonatal rat astrocytes cultured on surfaces coated either with PLL (**A, B**), Laminin (**C, D**), Fibronectin (**E, F**) or ECM gel (**G, H**). Cell nuclei are stained with Hoechst dye (blue). The scale bar represents 50 mm.
